# Editorial: Emerging Concepts on the NKG2D Receptor-Ligand Axis in Health and Diseases

**DOI:** 10.3389/fimmu.2020.00562

**Published:** 2020-04-07

**Authors:** Nadia Guerra, Lewis L. Lanier

**Affiliations:** ^1^Department of Life Sciences, Imperial College London, London, United Kingdom; ^2^Department of Microbiology and Immunology, University of California, San Francisco, San Francisco, CA, United States; ^3^The Parker Institute for Cancer Immunotherapy, San Francisco, CA, United States

**Keywords:** NKG2D, natural killer cells, tumor immunology, autoimmunity, cytomegalovirus (CMV)

The immune activating receptor NKG2D and its cognate ligands represent a fascinating immune recognition system, essential to the activation of innate and adaptive effector cells. It is a potent axis for the detection of danger recognized as MHC I-like self-molecules induced under stress conditions or during rapid proliferation. As such, it complements the recognition of non-self-antigens presented by classic MHC molecules, ensuring an early and efficient elimination of threats. The strength of the NKG2D pathway relies on its capacity to: (i) activate several immune effector cells due to the wide expression of the NKG2D receptor on lymphocytes, and (ii) distinguish diverse types of stress via multiple ligands displaying distinct affinities and levels of regulation.

The central dogma states that NKG2D engagement initiates or strengthens immune responses against infected and tumor cells by means of cytokine and chemokine secretion and direct cytotoxic activity. However, recently we have started to appreciate the multifaceted involvement of this pathway in immunity and how genetic polymorphism of the NKG2D-NKG2D ligand (NKG2DL) axis might explain the different and even contradictory outcome of NKG2D-driven responses observed across patients in certain disease context. Still, important gaps in knowledge remain regarding the regulation of human and mouse NKG2DL expression in space and time. The nature of ligand (binding affinity, membrane bound vs. soluble), the amount of ligand expressed and distribution at the membrane, and the cell types displaying ligands (tumor vs. normal tissue vs. stroma vs. immune cells) are likely to differentially impact on disease as it progresses and reciprocally be affected by it. Importantly, NKG2D ligand expression in healthy tissues is no longer anecdotal and it has become clear that NKG2D can contribute to the development of autoimmunity, highlighting the paradoxical outcome of NKG2D-NKG2DL engagement on disease progression (protective vs. detrimental), including in cancer.

This Research Topic gathers research articles and literature reviews from leaders in the NK and T cell biology fields highlighting recent advances on the expression and function of NKG2D and its ligands. Together, these 17 articles provide a timely survey of this recognition pathway as a critical immunologic component of health and disease to engage in further discussion and developments in basic and translational immunology.

## Expression and Regulation of NKG2D and NKG2D Ligands

There are eight human ligands for NKG2D with differential expression, affinity, and ability to be released as soluble and exosomes-bound forms, accounting for the difficulty to fully understand their unique and concomitant role in complex pathologies. These ligands are the MHC class I-related chain A and B (MICA, MICB) proteins and the six unique long 16 (UL-16)-binding proteins (ULBP1-6). Their counterparts in mice are the retinoic acid early inducible gene-1 (RAE-1α-ε), minor histocompatibility H60a-c, and murine UL16-binding protein-like transcript 1 (MULT1) proteins. The multiplicity of ligands warrants an efficient immune recognition of cell extrinsic and intrinsic danger signals.

In the last two decades, extensive knowledge uncovered the different layers of regulation of most ligands at the transcriptional, post-transcriptional, and post-translational levels. Yet, the lack of a holistic characterization of the mechanisms driving the heterogeneous expression of one or multiple ligands in different cell types, tissues, and disease contexts hampers their manipulation for therapeutic purposes. Zingoni et al. provide a comprehensive overview of the various levels of regulation of human and mouse NKG2D ligands, focusing on the specificity and redundancy of the molecular pathways able to simultaneously control the expression of several NKG2D ligands in normal and pathological settings. Further, they discuss the functional relevance of these distinct ligands or allelic variants in their ability to modulate NKG2D-mediated signaling consequently affecting target cell killing.

NKG2D polymorphism has not been extensively studied despites the rising number of allelic variants identified to date, including over 100 known allelic variants for MICA and MICB. Zuo et al. provide a state-of-the-art review on the polymorphism and functional features of human NKG2D ligands and their connections with different diseases. This comprehensive review compares and contrasts polymorphisms within MICA and MICB to the ULBP genes family and discusses the evolutionary pressures that have driven NKG2DL polymorphism. Notably, the authors discuss how viral infections may have acted as a selective force toward differential tissue-specific regulation of the ligands, allowing effective anti-viral control while limiting autoimmunity. The influence of polymorphisms on the NKG2DL expression, describing how MICA variants dictate not only the amount of protein expressed but also its subcellular location and shedding; the binding affinity to NKG2D and how it impacts effector functions are discussed. The clinical relevance of NKG2DL polymorphisms is considered in the context of autoimmune diseases, viral infections, cancer, and transplantation with the goal to translate that knowledge to target the NKG2D-NKG2DL axis for therapeutic purpose.

ULBP4 is the most polymorphic member of the ULBP family, yet the least characterized ligand for NKG2D. Zöller et al. discuss inconsistencies between the biochemical features of ULBP4 in the literature and publicly available databases and undertook to perform further biochemical and expression analyses in three tumor cell lines: C1R, HepG32, and HeLa. The authors confirmed several features of the mature ULBP protein in line with the prediction by Uniprot, e.g., histidine 31 occupies the first position in the mature ULBP4 glycoprotein, not as previously assumed with glycine 29, providing evidence of a recessed β1 strand of the ULBP4 α1 domain that may impact protein folding and function compared with other ULBP molecules. They characterized isoform 0 as a variant with a divergent carboxy-terminus that results in a shortened mature glycoprotein. To further study isoform 0, they generated a novel ULBP4 mAb and compared the specificity of staining with commercially available antibodies, raising concerns for potential artifactual staining with the latter. Interestingly, the authors show that the cervical carcinoma cell line HeLa does not express membrane bound ULBP4s yet contains a large amount of ULBP4 retained intracellularly. Finally, the authors show that the release of soluble ULBP4 is partly due to metalloproteases and possibly due to alternative splicing of the full-length isoform 0. These novel interesting findings will stimulate future efforts to address ULBP4 expression *in situ* and its physiological relevance in cancer immunity.

The presence of soluble NKG2D ligands in cord blood plasma (CBP) has been shown to be significantly higher than in adult plasma from healthy individuals. Here, Cox et al. examined the expression of different allelic variants for MICA and MICB in the coding and promoter regions in CBP samples and observed that common MICB alleles, such as MICB^*^005:02, resulted in increased concentration of soluble MICB (sMICB) with the promoter polymorphisms P2, leading to elevated expression of soluble MICB while the opposite was observed with promoter polymorphisms P9. *In vitro* assays showed that increasing concentration of soluble ULBP1 strongly associated with lower percentage of IFNγ+ NK cells among stimulated PBMC and moderately associated with CD107a+ CBP-derived NK cells. In contrast, the concentration of neither sMICA nor sMICB affected expression of CD107a whatever cell type studied. Yet, when samples were stratified according to homozygous MICB allele or promoter type, MICB^*^005:02 with MICB-P2, induced a lower % of IFN-γ+ NK cells compared with MICB^*^008 with MICB-P9 type indicating differential amount and inhibitory potential for sMICB. While sMICB and sULBP1 were detected in all CBP samples, sMICA was detected in only a third of the samples. Most sMICA detected in CBP corresponded to the Val/Val dimorphism known to be associated with a weak binding affinity for NKG2D. Cells exposed to plasma from UCB samples homozygous for MICA-129val showed significantly increased percentage of IFN-γ and CD107a compared with those from Met/Met or Val/Met samples indicating enhanced NK cell function. The authors propose a model that could contribute to fetal-maternal tolerance based on the presence of soluble NKG2D ligands in CBP. Future studies will need to address important pending questions including identifying the cell type(s) responsible for the presence of soluble ligands in CBP. It will be key to also demonstrate if and how various soluble ligand harboring different affinity (sMICB, sULBP1 vs. sMICA) may compete for NKG2D binding on fetal NK cells.

## NKG2D in Cytomegalovirus Infection

Cytomegalovirus (CMV) infection is a serious cause of morbidity in immuno-compromised transplant recipients with the majority of patients experiencing CMV re-infection or reactivation in the absence of anti-viral prophylactic treatment. Rohn et al. address this important topic in NK and T cell biology by determining the association of MICA and NKG2D polymorphisms with the clinical outcome of CMV. Both univariate and multivariate analyses of three functional SNPs in a cohort of 181 kidney donor-recipients' pairs revealed that the MICA rs2596538 GG donor genotype—known to be associated with elevated MICA expression- is significantly less frequent in patients displaying CMV disease during the first year after kidney transplant. Hence, they identify the G allele status as a protective prognostic determinant for CMV disease and the control of CMV viremia. Along with NK cells, CMV-specific CD8^+^T and CD4^+^T cells may be involved in controlling viral replication in an NKG2D-dependent manner. Future studies should aim to address the functional relevance and collaborative nature of these cell types, as well as the influence of soluble ligands during CMV re-infection or reactivation in transplanted patients.

With the goal to induce a strong and persistent immune response against CMV, Hiršl et al. explored the idea of developing a CMV vaccine vector expressing NKG2D ligands to generate protective T cell Immunity. The present studies are built upon previous work from the Jonjic lab where mouse CMV (MCMV) engineered to express RAE-1 in place of immune evasion gene m152 was proven to be highly attenuated yet retained the ability to induce a strong CD8^+^ T cell response *in vivo*. Here, they replaced m145 with MULT-1, a higher affinity ligand for NKG2D than RAE-1, and showed that MULT-1-MCMV replication in various infected tissues was attenuated in an NK cell- and NKG2D-dependent manner. It could elicit a robust adaptive response when transferred into immunocompromised mice. Interestingly, the authors detected the presence of anti-CMV antibodies in infected mice and showed that protective immunity can be transferred to the offspring of immunized females. Hence, the ability for such attenuated virus to induce a durable CD8^+^T cell memory response and a humoral response that can cross the placental barrier are critical features for its use as a vector vaccine. Safety concerns require further testing addressing whether attenuated virus can persist in the host and potentially be reactivated in immune-compromised patients, such as transplant recipients.

## NKG2D-NKG2D Ligands in Inflammation and Autoimmunity

Various sources of stress can induce or upregulate NKG2D ligands in healthy tissues, including immune cells, in the context of inflammation. Four complementary review articles address an emerging topic in the function of NKG2D-NKG2DL interactions as a regulatory axis of immune responses broadening our views on the potential mechanisms accounting for the conflicting role for NKG2D reported in disease settings.

Trembath and Markiewicz review the growing literature regarding NKG2D ligand expression on healthy immune cells, including on activated T cells, B cells, NK cells, and myeloid cells. The discussion encompasses studies reporting opposing effects of ligand induction on immune cells, either killing, suppressing, and/or stimulating the proliferation and functional activities of NKG2D-expressing cells, with evidence of reciprocal effects during cell-cell interactions. They discuss how the “immunological multi-purposing” of NKG2D-NKG2DL interactions can fine-tune immune responses toward different biological outcome in immune homeostasis, tumor immunity, and autoimmune diseases. More recently, the authors further reported the constitutive expression of ULBP-4 on healthy monocytes and resulting effect in downregulating NKG2D expression on NK cells (1).

Wensveen et al. further discuss the role of NKG2D in controlling the activation thresholds of T cells, NK cells, and B cells. They examine evidence supporting that depending on the intensity and duration of ligand engagement, NKG2D can mediate both stimulatory and inhibitory signals impacting on NK cell development and education. The authors further discuss the anergy in T cells and NK cells induced by chronic NKG2D stimulation and how it can be overcome in a sufficiently inflammatory environment. Finally, they discuss unexpected findings by their group demonstrating that the lack of NKG2D alters mouse B1a cell development at an early pro-B progenitors and reduce the anti-microbial activity of this subset, which are mainly present in the peritoneal and pleural cavities of mice. Further studies are required to support the hypothetical existence of NKG2D signaling in an early progenitor common to NK cells and B cells and consequent impact on gene expression relevant to lineage commitment at later stages of B cell development.

Stojanovic et al. provide an extensive review on NKG2D ligand expression on immune cells with a particular focus on myeloid cells, discussing evidence of NK cell crosstalk with dendritic cells and macrophages. The authors examine the data generated from different types of infectious agents including bacteria, viruses, and parasites that cause NKG2DL expression in myeloid cells, likely through TLR signaling. They highlight several *in vitro* studies reporting that other factors produced by stromal, parenchymal, and immune cells in response to infection upregulate NKG2D and its ligands, contributing to a crosstalk between myeloid cells and NK cells. Besides infections, the authors also discuss evidence for NKG2DL expression on myeloid cells infiltrating tumors and inflamed normal tissues under autoimmune attack from several clinical studies, as well as in experimental models. The nature of the NKG2DL that functionally impact an NKG2D-mediated crosstalk of myeloid cells and NKG2D+ lymphocytes and its outcome *in vivo* remains nonetheless understudied. Whether ILC1 and ILC3 via NKG2D also display the ability to communicate with myeloid cells under cellular stress is not known.

Besides immune cells, ligands for NKG2D are detected in healthy tissues under stress. Babic and Romagnani compare and contrast the literature on the role of the NKG2D-NKG2DL pathway in chronic inflammation and autoimmunity, discussing Type 1 diabetes, multiple sclerosis, Experimental Autoimmune Encephalomyelitis (EAE), Rheumatoid Arthritis (RA), celiac and Inflammatory Bowel diseases (IBD). They underline the presence of IL-15 as a common feature in most of these pathologies in priming CD4^+^T cells and CD8^+^T cells into cytotoxic cells and in driving the upregulation of NKG2D on those cells, as well as increasing NKG2DL expression on epithelial cells. Of importance, they discuss the enrichment of IL-17-producing cells among NKG2D^+^CD4^+^ T cells when compared to NKG2D^−^ CD4^+^ T cells in patients with Crohn's disease and in a model of colitis. With regards to intestinal inflammation, Hosomi et al. discuss novel findings from their group that uncovered the implication of endoplasmic reticulum (ER) stress as a novel mechanism for selective expression of the mouse NKG2D ligand MULT-1 on epithelial cells. Whether this is also the case in other autoimmune disease is not known, yet Babic and Romagnani highlight that the expression of MULT-1, along with RAE-1, in activated mouse oligodendrocytes led to their susceptibility to killing by activated CD4^+^ T cells enriched for the expression of NKG2D. Finally, the authors highlight that these important considerations led to the development of strategies aiming to block NKG2D-mediated activation to effectively reduce pathogenesis, as tested in animal models of RA, EAE, and IBD and currently in phase 2 clinical trials in patients with active Crohn's disease.

Similar to the gut, the liver is a tolerogenic organ that harbors a large amount of innate lymphoid cells and under constant exposure to pathogens. One particular feature of the liver is the elevated presence of invariant Natural Killer T cells (iNKT), which constitute the main immune cell type residing in this tissue in mice. On this topic, Dulaimi et al. demonstrated the contribution of NKG2D on iNKT cells in liver inflammation. They employ a model of Con-A-induced hepatitis in NKG2D wild-type (WT) vs. deficient (knockout, KO) mice to assess the relevance of the NKG2D-NKG2DL pathway on iNKT cells. The authors showed an upregulation of RAE-1 on hepatocytes upon Con-A treatment associated with an increased production of IFNγ, TNFα, and IL-4 in liver iNKT cells of NKG2D-WT mice compared to the KO group. One key finding is that antibody-mediated NKG2D blockade ameliorates disease by reducing liver injury and increasing mouse survival, which correlated with reduced cytokine production and FASL expression by iNKT cells. Increased survival due to NKG2D blockade was not to the level comparable to CD1d-deficient mice, however, suggesting additional pathway(s) by which iNKT cells drive liver damage in this model. An added value of this work is the characterization of thymic and peripheral iNKT cells in NKG2D-deficient mice showing that they are phenotypically and functionally similar to wildtype mice. The literature on NKG2D in iNKT cells is scarce (2), these valuable studies clearly demonstrate the impact of NKG2D in NKT cell activation upon liver inflammation. Yet, the actual contribution of NKG2D on iNKT cells as opposed to NK cells and other T cell subsets in this model remains to be determined. Notably, these data further probe the deleterious effect driven by NKG2D in inflammation, feeding the model that NKG2D-mediated liver damage could contribute to tumor development as discuss by Sheppard et al. in the next section.

## NKG2D in Cancer Immunity

While the NKG2D-NKG2DL axis is recognized as an attractive target for immunotherapy against cancer and inflammatory disorders—more information is needed to optimally design and tailor strategies that either boost or block this peculiar pathway. Hosomi et al. review the mechanisms involved in the upregulation of NKG2DL expression in cells upon genotoxic stress. These include activation of the DNA damage pathway through the ATM or ATR protein kinases and through the stimulator of interferon genes (STING)-dependent pathway upon accumulation of cytosolic DNA. Less studied is the role of the carcinoembryonic antigen-related cell adhesion molecule 1 (CEACAM1) in tumor immunity. The authors discuss their recent findings along other groups' studies on the mechanisms that suppress NKG2D-NKG2DL-mediated killing of cancer cells. On tumor cells, CEACAM1 was shown to prevent NKG2D ligand cell surface expression via intracellular retention as an incompletely glycosylated protein. On NK cells, co-engagement of NKG2D and CEACAM1 suppresses NKG2D-mediated signaling in a SHP1-dependent manner, hence reducing NK cell cytotoxicity against CEACAM1-expressing tumor cells. These data evidence the impact of CEACAM1 as a negative regulator of NKG2D ligands on cancer cells.

The normal liver contains a large population of resident lymphocytes largely enriched in NK cells, yet it is the site of a number of ineffectively controlled chronic infections, of primary hepatocellular carcinoma (HCC), and metastasis from colorectal cancer (CRC). Easom et al. studied NK cells in HCC and secondary liver tumors arising from CRC metastasis and evidenced surprisingly high frequencies of NK cells infiltrating tumors—a large majority of which displaying a liver resident phenotype (CXCR6^+^CD69^+^). Intratumor NK cells displayed fairly high levels of cell surface NKG2D, albeit lower compared to NK cells located in healthy adjacent tissues. It would be very interesting to assess whether the presence and amount of NK cell infiltrating liver tumors correlates with disease prognosis in HCC and metastatic CRC patients. Further, the authors compared the level of granzyme B (GzB) expression between healthy peripheral NK, intrahepatic NK cells [including liver resident (lr) NK and non-lrNK cells] and tumor-infiltrating NK cells. They show a heterogeneous expression of GzB^hi^ and GzB^low^ expressing cells in intrahepatic and tumor-infiltrating NK cells which contrasted with the homogeneous GzB^hi^ level detected in peripheral NK cells. The percentage of IFN-γ-producing NK cells was not significantly different in tumor vs. healthy liver, yet significantly lower than in circulating NK cells. Further studies will be needed to directly link the reduced NK cell functionality with NKG2D and NKG2DL expression in HCC patients. The authors also performed overnight coculture experiments of healthy intrahepatic NK cells with a MICA+ HCC-derived cell line and showed the ability of the HCC cell line to downregulate NKG2D and reduce NK cell activation. This effect was cell-cell contact dependent and NK cell activation could be restored by exposure to IL-15. Future work employing advanced cytometry to assess intertumoral NK cell functional heterogeneity (3) and autologous tumor cells in functional assays will be key, along with developing ways to use IL-15 to potentiate NKG2D-mediated response *in situ* with limited toxicity toward healthy hepatocytes.

The persistence of chronic inflammation is well-established as a contributing factor for tumor development, especially in the liver. The role of NKG2D-NKG2DL in this process is currently understudied, relying on a few clinical and experimental evidence and conflicting with its canonical function in tumor suppression. Sheppard et al. discuss the literature regarding the prognostic value for NKG2D ligands in various cancer types as well as experimental evidence supporting a tumor-promoting effect of NKG2D and CD8^+^T cells in models of liver injury and cancer. A role for NK cells in HCC development is supported by recent evidence that the accumulation of CD49a^+^ NK cells in liver tumors associates with tumor progression and poor clinical outcome (4). Sheppard et al. discussion stems from their recent finding that NKG2D promotes rather than delays tumor progression in a long-term model of chemically-induced liver cancer (5). These studies showed that HCC progressed more rapidly in NKG2D-WT than NKG2D-KO mice leading to increased tumor burden and tumor grade in a tissue that expresses elevated levels of membrane-bound NKG2DL. The authors propose that at early stages of tumor development, the expression of NKG2D ligands on precancerous lesions facilitate tumor clearance, however, over time, chronic activation of NKG2D^+^ effector cells residing in the healthy liver combined with a partially or fully impaired NKG2D-dependent tumor rejection within the tumor bed could favor tumor progression. They postulate that in settings of chronic inflammation, NKG2D sustains a feedback loop of tissue injury and repair in the healthy tissue adjacent to the tumor which exacerbates the formation of inflammatory niches favorable for neotumor development. This hypothesis awaits to be supported by further demonstration in different models of cancer and by the identification of the cell type(s) contributing to the dual function of NKG2D. These findings stimulate further discussion and encourage careful considerations regarding the need to stratify patients that may benefit from NKG2D-based therapies.

Beyond NK cells and CD8^+^T cells, the NKG2D-NKG2DL system is highly relevant to γδT cell-based therapy. Bhat et al. examined the effect of six pharmacological inhibitors of the epigenetic modifier histone deacetylases on the expression and shedding of several NKG2D ligands in a pancreatic and a prostate carcinoma cell line. Exposure to Valproic acid (VPA) significantly increased the cell surface expression of MICA, MICB, and ULBP2, but not ULBP1, and their release as soluble forms, highlighting discrepancy with data obtained in other cancer types. Decreased NKG2D expression at the protein and RNA levels were observed in activated Vδ2 T cells, but not PBMC when treated with VPA and co-cultured with tumor cell lines, albeit this did not affect their capacity to degranulate. The authors also established a flow cytometry-based method to distinguish high vs. low levels of histone acetylation (H3K9c) associated with changes in the relative distribution of Vδ2 T cell subpopulations when treated with VPA. While shedding further light on the epigenetic regulation of NKG2D and its ligands in γδ T cells and tumor cells, respectively, the biological significance of the findings remains to be demonstrated using fresh tumor tissues from prostate and pancreatic cancer patients to appreciate the implications in forecasting VPA treatment in combination with γδ T cell-based therapy.

With immunotherapy rapidly expanding as a novel option in the treatment landscape of several cancer types, two comprehensive reviews discuss the rationale for the development of immune-based therapies exploiting the NKG2D-NKG2DL axis. Frazao et al. review clinical studies that describe the role of the NKG2D-NKG2DL axis in cancer immunosurveillance indicating how NKG2D and NKG2D ligand polymorphisms affect cancer development and the patient response to chemotherapy. The authors review clinical studies reporting that low NK cell numbers within tumors associate with bad prognosis and that the expression profile of NK cell receptors strongly influence prognosis and disease outcome. Interestingly, they point out the exception of patients with chronic myeloid leukemia (CML) for whom NK cell counts are a significant predictive parameter for relapse after imatinib discontinuation. They further discuss the potential of Chimeric Antigen Receptor (CAR)-NK cells in complement or alternative to CAR-t cells, focusing on the benefits of employing NKG2D-CAR, an approach currently under trial.

Schmiedel and Mandelboim review focuses on the various mechanisms of NKG2D ligand regulation and how these can impact tumor evasion. The authors also discuss current approaches that target NKG2D-NKG2DL expression for cancer immunotherapy including approaches that directly or indirectly interfere with the release of soluble ligands in advanced cancer and those that aim to boost anti-tumor responses to overcome the immunosuppressive tumor milieu.

## Conclusions

Overall, these articles illustrate key findings on the expression and function of the NKG2D-NKG2DL axis and highlight current challenges in the field. Outstanding fundamental questions remain regarding the role of NKG2D in ILCs, how chronic NKG2D signaling may affect NK and T cell metabolic status potentially leading to a stage of “exhaustion,” and how NKG2DL might play a role in cell recruitment to inflamed tissues. Further work is also needed to generate a holistic view on the evolution of NKG2DL expression with disease stepwise progression over time and their prognostic and/or predictive value in cancer patients.

Several approaches are underway to enhance NKG2D-mediated tumor recognition *in situ* and upon adoptive transfer of NK cells or T cells to treat cancer patients. Concomitant with blocking inhibitory checkpoints and increasing NK cell longevity, these approaches hold great promise for an effective targeting of solid tumors with no tumor-associated antigens identified. The three main challenges yet to be overcome are how to deal with low level of ligands on advanced cancer cells and cancer stem cells (6), and how to favor migration of adoptively transferred cells to the tumor microenvironment rather than residing in the healthy tissues, hence limiting toxicity and autoimmunity.

## Author Contributions

NG wrote the editorial. LL edited the editorial.

### Conflict of Interest

LL and the University of California, San Francisco have licensed intellectual property rights regarding NKG2D for commercial applications. NG received funds from AstraZeneca. The funder was not involved in the writing of this editorial or the decision to submit it for publication.
